# Comparative Transcriptome and Microscopy Analyses Provide Insights into Flat Shape Formation in Peach (*Prunus persica*)

**DOI:** 10.3389/fpls.2017.02215

**Published:** 2018-01-04

**Authors:** Jian Guo, Ke Cao, Yong Li, Jia-Long Yao, Cecilia Deng, Qi Wang, Gengrui Zhu, Weichao Fang, Changwen Chen, Xinwei Wang, Liping Guan, Tiyu Ding, Lirong Wang

**Affiliations:** ^1^The Key Laboratory of Biology and Genetic Improvement of Horticultural Crops (Fruit Tree Breeding Technology), Ministry of Agriculture, Zhengzhou Fruit Research Institute, Chinese Academy of Agricultural Sciences, Zhengzhou, China; ^2^The New Zealand Institute for Plant & Food Research Limited, Auckland, New Zealand

**Keywords:** fruit shape, peach, transcriptome, cell number and size, fruit diameter, fruit development

## Abstract

Fruit shape is an important external characteristic that consumers use to select preferred fruit cultivars. In peach, the flat fruit cultivars have become more and more popular worldwide. Genetic markers closely linking to the flat fruit trait have been identified and are useful for marker-assisted breeding. However, the cellular and genetic mechanisms underpinning flat fruit formation are still poorly understood. In this study, we have revealed the differences in fruit cell number, cell size, and in gene expression pattern between the traditional round fruit and modern flat fruit cultivars. Flat peach cultivars possessed significantly lower number of cells in the vertical axis because cell division in the vertical direction stopped early in the flat fruit cultivars at 15 DAFB (day after full bloom) than in round fruit cultivars at 35 DAFB. This resulted in the reduction in vertical development in the flat fruit. Significant linear relationship was observed between fruit vertical diameter and cell number in vertical axis for the four examined peach cultivars (*R*^2^ = 0.9964) at maturation stage, and was also observed between fruit vertical diameter and fruit weight (*R*^2^ = 0.9605), which indicated that cell number in vertical direction contributed to the flat shape formation. Furthermore, in RNA-seq analysis, 4165 differentially expressed genes (DEGs) were detected by comparing RNA-seq data between flat and round peach cultivars at different fruit development stages. In contrast to previous studies, we discovered 28 candidate genes potentially responsible for the flat shape formation, including 19 located in the mapping site and 9 downstream genes. Our study indicates that flat and round fruit shape in peach is primarily determined by the regulation of cell production in the vertical direction during early fruit development.

## Introduction

Fruit development is mainly controlled by two processes, cell division and cell expansion (Coome, 1976) that directly affect fruit cell number, cell size, and fruit size. The pattern of fruit cell distribution and expansion further affects fruit shape (Chusreeaeom et al., 2014). In tomato, various fruit shape mutants have been collected and used for map-based cloning of the gene underpinning the mutant phenotype. These genes include *FASCIATED* (*FAS*) (Cong et al., 2008) and, *LOCULE NUMBER* (*LC*) (Munos et al., 2011) conferring flat fruit by increasing locule numbers; *SUN* (Xiao et al., 2008) and *OVATE* (Liu et al., 2002) causing fruit elongation (Rodriguez et al., 2011) by increasing and reducing cell numbers in the vertical and transversal direction of the fruit, respectively (Wu et al., 2011). A novel tomato mutant (*Slelf1*) exhibits elongated fruit shape caused by increased cell layers in the proximal region of the ovary (Chusreeaeom et al., 2014). In peach, cell number is the controlling factor in fruit size between small- and large-fruited peach cultivars (Scorzal et al., 1991). Cell number is one of the most vital aspects that determine fruit development, including fruit shape and size, and has been reported in many fruit crops, such as apple (Denne, 1960), strawberry (Cheng and Breen, 1992), melon (Higashi and Hosoya, 1999), olive (Rapoport and Costagli, 2004), sweet cherry (Olmstead et al., 2007) and tomato (Bertin et al., 2003; Bertin et al., 2009).

Besides cell proliferation, cell expansion also plays an important role in fruit development. Cell expansion often occurs during late stages of fruit development when cell division has completed (Gillaspy et al., 1993). In apple, fruit size is positively correlated to fruit cell size because cultivars with large fruit has much larger fruit cell size than wild small fruit accessions (Yao et al., 2015) and a sport mutation has enlarged fruit by enhancing fruit cell expansion (Malladi and Hirst, 2010). In addition to fruit trees, grain size and shape have also been found to be controlled by *WTG1* (WIDE AND THICK GRAIN 1) through regulating cell expansion (Huang et al., 2017). Positive relationships between fruit size and cell size were reported among five fruit genotypes in apples (Harada et al., 2005), as well as in tomatoes (Cheniclet et al., 2005). However, there are cases where fruit size is not strongly associated with fruit cell size but with cell number in peach (Scorzal et al., 1991), strawberry (Cheng and Breen, 1992), pear (Zhang et al., 2006) and sweet cherry (Olmstead et al., 2007). Therefore, the cellular mechanisms controlling fruit development vary in different fruit crops. Investigating the cellular differences between flat and round peaches will enhance our understanding of fruit development.

Fruit shape is critical to the appearance of the fruit and affects the marketability of fruit crops. For peach (*Prunus persica*), the typical fruit shape is round, but more and more peach cultivars producing saucer-shaped fruit have been bred in recent years (Iglesias, 2013). The flat peaches (*P. persica* var. *platycarpa*) are called ‘Pantao’ in China and ‘Saturn peach’ or ‘donut peach’ in western countries. The flat fruit is a mutant of round fruit and is controlled by a single dominant gene as first described by Lesley (1940). This gene is mapped to the distal part of chromosome 6 (Lesley, 1940; Dirlewanger et al., 1998, 2006; Picañol et al., 2013; Micheletti et al., 2015; Cao et al., 2016; López-Girona et al., 2017). Two different candidate genes have been suggested for controlling this trait (Cao et al., 2016; López-Girona et al., 2017), indicating that further work is required to unequivocally indentify this gene.

For peaches, the cellular basis of fruit shape variations remains unknown, although the cellular basis of different fruit size is determined (Scorzal et al., 1991). Transcriptomic analysis has not yet been used to filter flat peach candidate genes although it is an effective method to screen the candidate genes at a genome wide scale (Zhou et al., 2014; Kanjana et al., 2016; Czemmel et al., 2017; Estrada-Johnson et al., 2017; Liu et al., 2017; Xu et al., 2017). In the present study, we aimed to illustrate the differences in fruit cell number and size, and in gene expression pattern between flat and round fruit by using histological sections and transcriptomic analyses. We found that peach fruit shape was primarily determined by the difference in cell number along the vertical direction of the fruit and identified 28 differentially expressed genes (DEGs), including 19 DEGs within the map region of flat fruit locus and 9 having potential functions in cell number regulation in the whole genome. These results would guide further studies to identify the flat shape gene and improve our understanding of fruit development in peach, as well as in other fruit trees.

## Materials and Methods

### Plant Materials and Sample Collection

**FIGURE 1:**
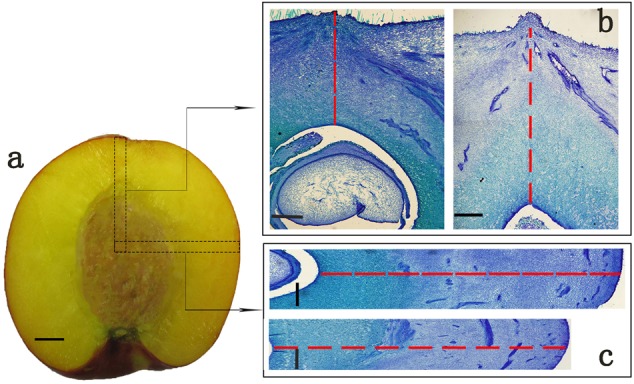
The typical target regions of peach fruit for microscopy analysis. **(a)** Fruit parts marked with black dotted boxes in vertical and transversal direction were selected for paraffin section analysis. **(b,c)** Are paraffin sections corresponding to the regions in transverse and vertical direction. **(b)** Stands for sections in vertical direction of flat (left) and round (right) shaped peach, while **(c)** stands for sections in transverse direction of flat (up) and round (down) shaped peach. Cell number was calculated along the red dashed lines in one straight single-cell line in **(b,c)**. Cells calculated in this way were regarded as cell number in vertical or transversal diameter. Scale bar = 1 cm **(a)**, and 500 μm **(b,c)**.

Four peach cultivars were selected in this study, including two round ones ‘Zhong Nong Jin Hui’ (JH) and ‘Zhong Tao Hong Yu’ (HY), and two heterozygous flat ones ‘Xin Hong Pan Tao’ (XH) and ‘Zao Huang Pan Tao’ (ZH). They are widely cultivated in China with similar harvesting date but not directly related, thus can represent for flat and round peaches (**Supplementary Figure S1**). All trees used in this study were grown at Zhengzhou Fruit Research Institute, CAAS. Fruit diameters (including vertical diameter and cheek diameter) of six fruit per cultivar were measured from full bloom to fruit maturity at 0, 10, 15, 20, 25, 30, 35, 40, 45, 55, 65, and 75 DAFB (day after full bloom), using a digital caliper. Fruit samples at 0 DAFB were collected from almost 200 full bloom flowers by removing the petals and styles. Vertical diameters were measured from the top to bottom of the fruit in the vertical axis and cheek diameters were between the cheeks of peach. In addition, six fruit per cultivar were used for microscopy analysis to determine histological differences. For microscopy, fruit samples were collected at 0, 15, 25, 35, 55, and 65 DAFB and fixed in FAA (75% ethyl alcohol, 5% formalin, 5% acetic acid), then stored at room temperature until further analysis. To measure cell number and cell size of the two different fruit shape types, we selected regions in vertical sections from the middle of the fruit kernel to the top in the vertical direction, and the middle of the fruit kernel to fruit cheek side in transversal direction (**Figure 1a**). Because of fruit stone hardening, samples prepared for microscopy analyses contained exocarp, mesocarp, and endocarp before fruit pit hardening (35 DAFB) and exocarp, mesocarp merely for the late two periods. For comparative transcriptome analysis, HY and ZH fruit samples were collected at 0, 15, and 65 DAFB for RNA sequencing with three biological replicates.

### Paraffin Section Analysis

Samples were removed from FAA and rinsed thoroughly in deionized water. Then, the samples were dehydrated using gradient ethanol, transparentized with xylene, and embedded in wax finally. For determining the cellular basis of flat and round shape formation, we focused on the vertical and transversal fruit development. For early fruit samples, section thickness was from 10 to 15 μm, whereas that for fruit samples collected at late development stages was from 15 to 30 μm. All sections were rehydrated and stained using aqueous toluidine blue (pH 7.0) (Yao et al., 2015). Images were captured with a light microscope (Olympus) fitted with a camera (DP71; Olympus).

### Analysis of Cell Number and Area

To determine the cell number in the target regions (**Figures 1b,c**), we counted all cells using a light microscope. Six samples per cultivar were counted for each development stage. The cells were counted one by one along vertical or transversal axis through the eyepiece of a light microscope. Here, we provided a model for cell number calculating. The cells along the red dotted line in **Figures 1b,c** were counted in one straight single-cell line direction. The cell number that calculated in this way was regarded as cell number in vertical or transversal axis in this study. Cell area measurements were made using the ImageJ software^1^. Because of pit hardening, the target regions of early fruit contained parts of endocarp, whereas regions of late development fruit did not. Therefore, the cell number of sections of the late fruit developmental period was calculated by adding the cell number of endocarp for each cultivar.

### Statistical Analysis

For microscopy analysis, the data was subjected to analysis of variance using the SAS general linear model procedure with the variance for subsamples used as the error term (SAS Institute, Cary, NC, United States). The SAS correlation procedure was also used for other appropriate analysis.

### Validation of Flat Shape Genetic Marker

To validate the candidate gene for flat shape, the DNA marker PC4 was amplified using primer pairs FlatIn_F and IndelS_R as described in a previously published paper (López-Girona et al., 2017) from 72 peach cultivars that included 42 heterozygous and 1 homozygous flat cultivar which aborted at early fruit development, and 30 round cultivars (**Supplementary Table S1**). The 3730XLDNAanalyzer equipment (ABI, United States) was used for genotyping.

### Total RNA Extraction

Total RNA was extracted from 18 peach samples that represent for three biological replicates of two peach cultivars at three development stages (named HY1-1, HY1-2, HY1-3, HY2-1, HY2-2, HY2-3, HY3-1, HY3-2, HY3-3, ZH1-1, ZH1-2, ZH1-3, ZH2-1, ZH2-2, ZH2-3, ZH3-1, ZH3-2, ZH3-3), using the RNAprep Pure Plant Kit (Tiangen, Beijing). RNA integrity was confirmed by 1% agarose gel electrophoresis. After digestion with DNase I at 37°C for 30 min to remove DNA residue, RNA quality and concentration were measured using an the NanoPhotometer spectrophotometer (Thermo, United States).

### Library Construction and Transcriptome Sequencing

Total RNA of 20 μg from each of the 18 RNA samples was sent to Novogene (Beijing, China) for construction of RNA-seq libraries and sequencing. The mRNA was purified from the total RNA using the Oligotex mRNA Midi Kit (Qiagen, Beijing), and assessed for quality using the Agilent Technologies 2100 Bioanalyzer (Agilent, United States). The mRNA was then broken into short fragment (approximately 300 bp). First and second strand complementary DNA (cDNA) was synthesized using a cDNA Synthesis System kit (TOYOBO, Japan) using random hexamer-primer, following the manufacturer’s protocol. Then double-strand cDNA were purified and adapters were ligated to the short fragments. The constructed RNA libraries were sequenced on the Illumina HiSeq^TM^ 2000 platform in paired-end (PE) mode.

### RNA-Seq Data Analysis

Reference peach genome V1.0^2^ and annotation files were downloaded from GDR website^3^. The index of reference genome was generated by hisat2-build script. RNA-seq data was filtered to remove the low-quality reads using FASTX toolkit^4^ based on the Q20 value per base. Cleaned reads were mapped to the reference peach genome using Hisat2 software (Kim et al., 2015). The alignments were compressed and sorted into bam files using Samtools (Li et al., 2009). Based on the alignments, transcript abundances were estimated and transcript assembly was performed using the Stringtie (Pertea et al., 2015) program. DEG analysis was carried out using the R package ballgown (Frazee et al., 2015).

### Validation of RNA-Seq Data by Quantitative RT-PCR

The expression level of selected DEGs identified from RNA-seq data were validated by qRT-PCR using the cDNA samples used for RNA-Seq library construction. The primer pairs (**Supplementary Table S2**) were designed with primer-blast program from NCBI^5^. The qRT-PCR was performed by Roche LightCycler480 with the following procedure: 95°C for 5 min, followed by 45 cycles at 95°C for 10 s, 60°C for 10 s and 72°C for 20 s. The house-keeping gene RP-II was used as an internal control (Cao et al., 2016). The relative expression level was calculated by the 2^-ΔΔC_T_^ method (Livak and Schmittgen, 2001).

### IAA Measurement in Flat and Round Peach

Plant materials used for IAA measurement were the same as materials for RNA-seq. To determine the IAA content in each sample, the HPLC-MS/MS system (HPLC, Shim-pack UFLC SHIMADZU CBM30A system^6^; MS, Applied Biosystems 4500 Triple Quadrupole^7^) analyses were carried out.

## Results

### Differences in Fruit Diameter and Cell Number between Flat and Round Peaches

**FIGURE 2:**
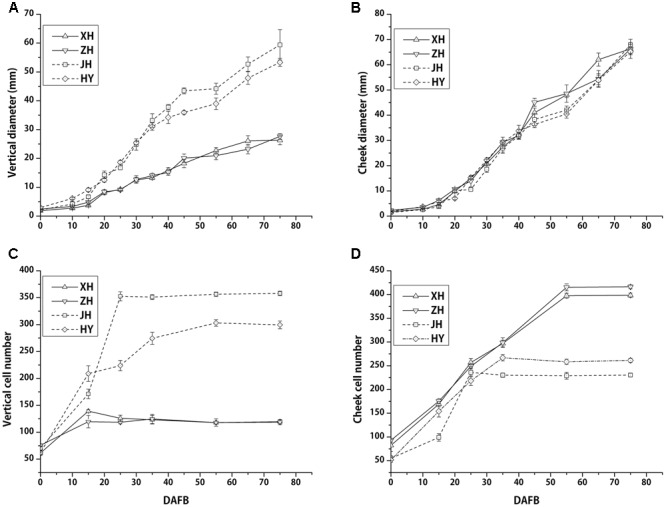
Fruit diameter and cell number among four different cultivars (two flat and two round peaches) during fruit development. Fruit vertical diameters of flat peaches are smaller than those in round ones from the early period of fruit development. Cell division in flat peaches ceases at almost 15 DAFB, while it continues in round ones until 35 DAFB **(A,C)**. Cheek diameters are similar among flat and round peaches **(B)**. Transversal cell numbers were higher in flat peaches than in round peaches **(D)**. Two solid and dashed lines stand for flat and round peaches, respectively. Each data point consists six data for each cultivar (*n* = 6) and the bars stand for SD.

After recording fruit vertical diameter both vertical and transverse directions, we found that vertical diameter of flat peach was much smaller than that of the round peach. This difference started from 15 DAFB and was present during entire subsequent development periods. The final vertical diameters of round shaped peaches were almost two times larger than flat ones, conferring obviously different shape phenotypes (**Figure 2A**). However, fruit diameter in the transversal direction was almost the same and increased at a similar speed between flat and round peaches from full bloom to fruit ripening (**Figure 2B**). Collectively, these data indicated that there were no significant differences between flat and round shaped peaches in fruit cheek diameter, whereas there was a difference in fruit vertical diameter.

In addition, cell number were counted across the vertical and transversal axis. In the vertical axis, cell number continued to increase from 0 to 35 DAFB in the two round peach cultivars, whereas it ceased to increase at an early fruit development stage (15 DAFB) in two flat ones (**Figure 2C**). The final cell number for the flat peach cultivars was approximately 2 to 3 times lower than that in the round ones. In the transversal axis, cell number stopped increasing at 35 DAFB in the round peach cultivars, while it continued to increase until 55 DAFB in the flat ones, which resulted in much more cells in the flat ones in this direction (**Figure 2D**). Cell size of flat peach is smaller than that in round peach in transversal axis, which results in the similar fruit cheek diameter (**Supplementary Figure S2**). Consequently, the reduction of cell number in the vertical diameter is one important factor which resulted in the limitation of fruit vertical development in flat peach cultivars.

### Correlation among Fruit Vertical Diameter, Cell Number, and Cell Size

**FIGURE 3:**
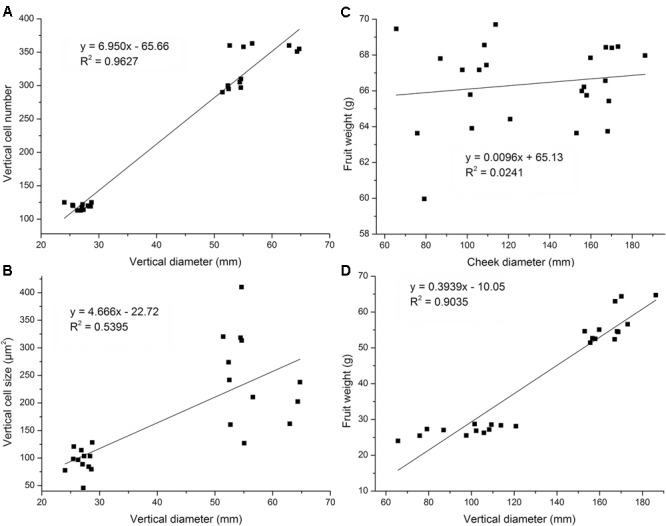
Linear regression analysis of fruit diameter, cell area, and cell number at fruit maturation stage. Linear regression analysis showed no strong linear relationship between cheek diameter and fruit weight **(B)** but a strong linear relationship between vertical diameter and fruit weight (*R*^2^ = 0.9627) **(A)**. Linear regression analysis showed no linear relationship between cheek diameter and fruit weight **(C)** but a strong linear relationship between vertical diameter and fruit weight (*R*^2^ = 0.9035) **(D)**. Four cultivars were used. Each cultivar was measured six times at fruit maturation stage (*n* = 6).

Cell number and cell size are two vital factors that facilitate fruit development. Herein, we analyzed the relationship between fruit vertical diameter and cell number and also analyzed the relationship between vertical diameter and cell size to determine the main causes that may be responsible for variation in flat and round fruit shape at maturation stage. A relative strong linear relationship between fruit vertical diameter and cell number was observed (*R*^2^ = 0.9627) which indicated that cell number was the main factor for fruit flat shape formation (**Figure 3A**). However, there was no strong linear relationship between cell size and fruit vertical diameter (*R*^2^ = 0.5395) (**Figure 3B**), which indicated that cell size contributed almost equally to fruit development, with cell area enlargement and vertical diameter increasing synchronously. Linear regression analyses were also performed for fruit weight vs. fruit vertical or cheek diameter. Strong linear relationship was found in the analysis of final fruit weight vs. vertical diameter (*R*^2^ = 0.9035), not in fruit weight vs. transversal diameter (*R*^2^ = 0.0241) (**Figures 3C,D**). This observation indicated that cell number, not cell size, was the factor that controlled variation in flat fruit shape.

In addition, we also analyzed the relationship between final cheek diameter and cell number or cell area with the examined cultivars. There were no differences in fruit cheek diameter between flat and round peaches. In transversal axis, no strong linear relationships were observed between fruit cheek diameter and cell number, as well as fruit cheek diameter and cell area (**Supplementary Figure S3**), which indicated that both cell number and cell size played important roles in fruit transversal development. Collectively, these results suggested that cell size may not play a vital role in the variation in flat and round fruit shape development in the vertical direction, whereas the reduction in cell number in the flat peach may be the key factor.

### Flat Shape DNA Marker

Two candidate genes for flat peach have been identified in previous studies, including *ppa003772m* (Cao et al., 2016) and *ppa025511m* (López-Girona et al., 2017). The marker in *ppa003772m* and *ppa025511m* has been verified in 474 and 178 peach accessions, respectively, with 100% accuracy rate in the previous studies (Cao et al., 2016). However, the marker in *ppa025511m* did not show a 100% association with flat fruit phenotype in this study when 72 peach cultivars were genotyped using the same primers as described in López-Girona et al. (2017). The genotype of 18 cultivars were not consistent with their fruit shape phenotype, including 14 of the 42 heterozygous flat cultivars, one homozygous flat peach (aborting at early fruit development) and 3 of the 30 round cultivars (**Supplementary Table S1**). The association rate was only 75%.

### Overview of RNA-Seq Data

A total of 67.4 Gb cleaned data was generated from the 18 cDNA libraries. The minimum number of reads per library was 38.7 million for ZH2-3 and the maximum number was 50.1 million for ZH2-2. The read length was 150 bp. Approximate 90% of the reads were mapped to the pear reference genome V1.0^8^ (**Table 1**).

**Table 1:** Mapping of RNA-seq reads from flat and round peaches at three developmental stages (0, 15, and 65 DAFB), against the peach reference genome.

Sample	Total reads	Mapped reads	Mapping rate	Properly paired rate	Multi-mapped
HY1-1	45145716	40905917	90.61%	83.94%	103930
HY1-2	43768758	39850342	91.05%	84.97%	69656
HY1-3	40917012	36979026	90.38%	83.45%	91808
HY2-1	44785292	40979054	91.50%	85.25%	89046
HY2-2	46678933	42626446	91.32%	84.94%	85800
HY2-3	49810673	45324327	90.99%	84.14%	97628
HY3-1	45573579	41476971	91.01%	84.01%	120526
HY3-2	41781704	37927029	90.77%	83.60%	109914
HY3-3	42526514	38009336	89.38%	80.92%	112400
ZH1-1	47028500	42758648	90.92%	84.43%	60742
ZH1-2	42608835	38626544	90.65%	83.72%	83768
ZH1-3	44555427	40468199	90.83%	84.08%	90272
ZH2-1	43792640	39795872	90.87%	83.97%	68972
ZH2-2	50069566	45490751	90.86%	83.87%	80284
ZH2-3	38745425	35283521	91.07%	84.32%	65964
ZH3-1	42767975	38442964	89.89%	82.11%	93512
ZH3-2	44916893	39067721	86.98%	79.80%	90576
ZH3-3	49153028	44245128	90.02%	82.67%	73732

### DEGs Analysis

**FIGURE 4:**
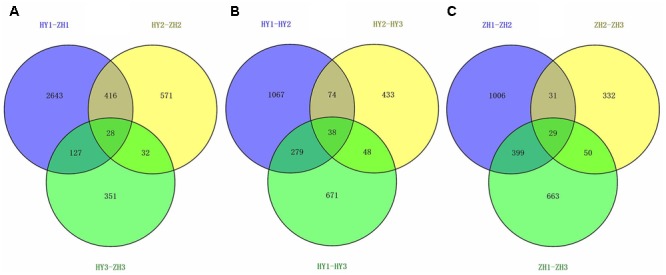
The venn diagrams of DEGs. **(A)** Venn diagram for HY1-ZH1, HY2-ZH2, and HY3-ZH3. **(B)** Venn diagram for HY1-HY2, HY1-HY3, and HY2-HY3. **(C)** V Venn diagram for ZH1-ZH2, ZH1-ZH3, and ZH2-ZH3.

Differential expression test was done between flat and round peach cultivars at three developmental stages (0, 15, and 65 DAFB) and 4165 DEGs were identified (**Supplementary Table S3**). There were 3231, 1049, 541genes identified as DEGs at the three developmental stages between flat and round peach with *P*-value < 0.05 (**Figure 4A**). Comparing the three developmental stages, much more genes (3231) were differentially expressed at full bloom date when the difference in fruit shape started to emerge. There were less DEGs at 65 DAFB between flat and round peach when fruits were close to maturity. In addition, we also analyzed DEGs among different stages in the same cultivar and compared them with each other in order to find some common DEGs. There were 1458 DEGs in HY1_vs_HY2, 593 in HY2_vs_HY3, 1036 in HY1_vs_HY3, 1465 in ZH1_vs_ZH2, 442 in ZH2-vs_ZH3, and 1141 in ZH1_vs_ZH3 (**Figures 4B,C**). And there were no common DEGs among them.

### Functional Annotation of DEGs at 0 DAFB

**FIGURE 5:**
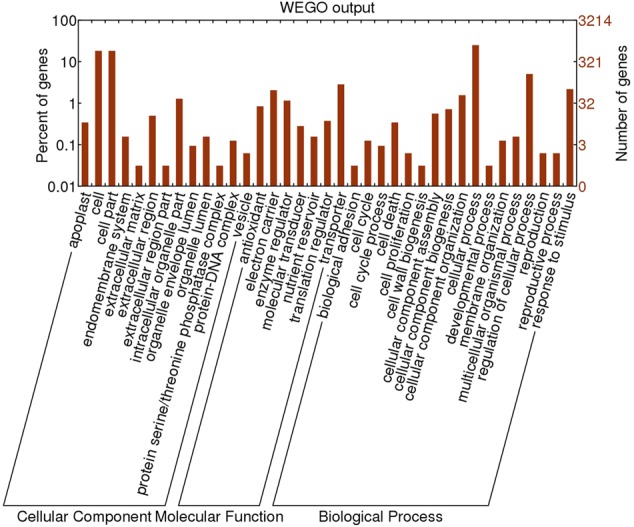
Gene ontology annotation of DEGs identified in comparative transcriptome analysis between flat and round peaches at 0 DAFB.

To identify genes important to fruit shape, we annotated the DEGs at 0 DAFB because full bloom is the earliest stage showing differences in cell number between flat and round fruits, thus is the critical time for identification of relevant genes. Based on GO (gene ontology) annotation, all the DEGs were clarified into three main categories, including cellular component, molecular function, and biological process (**Figure 5**). Most of DEGs belonged to cellular component and biological process, with each category possessing 13 and 17 terms. The most common terms were cellular process and processes related to cells in the biological process category. Genes related to cell cycle, cell proliferation, cell death, and cell wall biogenesis were also enriched. In the cellular component category, the main terms were cell and cell part. Overall, lots of DEGs were enriched in cell-related processes.

### Selection of Genes for Fruit Shape Variation

**FIGURE 6:**
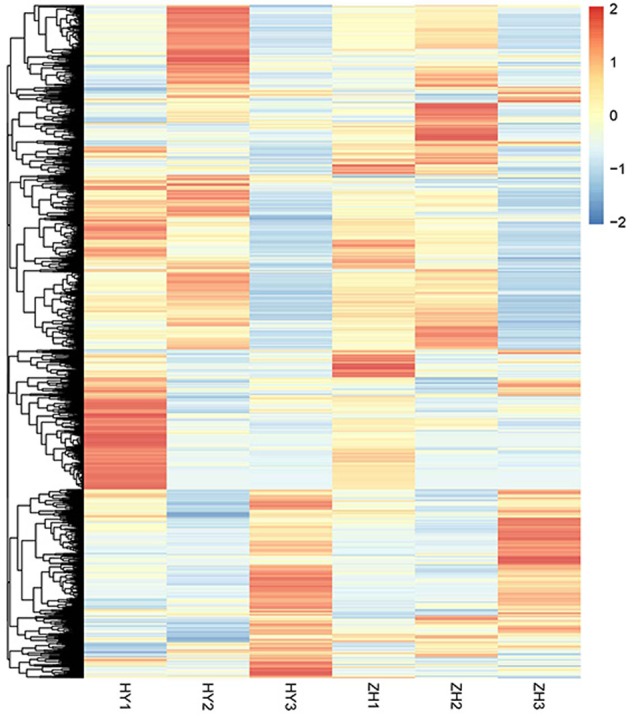
The expression level heatmap of all DEGs detected between flat and round peaches.

Using the FPKM (fragments per kilo-base per million mapped reads) value of 4165 DEGs, the heatmap was made to show relative gene expression patterns. From the heatmap (**Figure 6**), we found that some genes that were highly expressed in round or flat peach at 0 DAFB clustered together. The main factor leading to fruit shape variation in this study was the difference in cell number in vertical diameter between flat and round shaped fruit. Thus, genes that may be correlated with cell proliferation were further analyzed.

**FIGURE 7:**
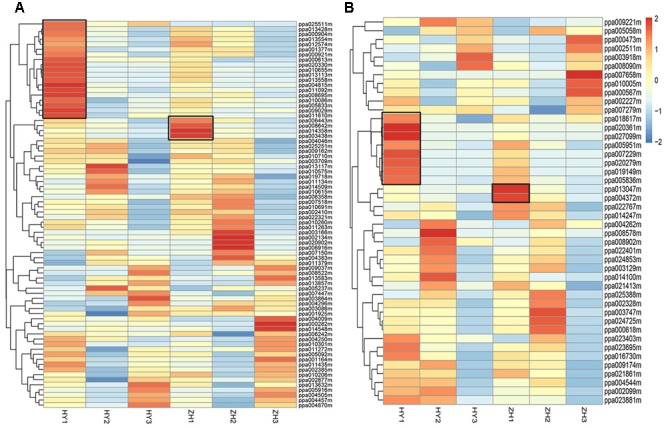
The expression level heatmap of selected DEGs. DEGs located within one Mega-base up- or down-stream of *ppa003772m* **(A)** and DEGs associated with cell number **(B)**. Genes associated with cell number were selected from GDR *Prunus persica* Pathway Search (https://www.rosaceae.org/). Black box shows the genes that had higher expression levels in round or flat peach and had higher expression levels at early fruit development stage than at the two late fruit development stages.

First, we selected 346 genes that were located within 1 Mb up- or down-stream of the main GWAS (genome wide association study) locus for fruit shape (Cao et al., 2016). Of these 346 genes, 76 were differentially expressed. According to expression pattern in the heatmap (**Figure 7A**), 23 DEGs were selected as candidates for the flat shape gene, including 19 highly expressed in round peach and 4 highly expressed in flat one at 0 DAFB.

Then, we selected 231 genes that were associated with cellulose biosynthesis, auxin biosynthesis, cytokinin biosynthesis, gibberellin biosynthesis, cell cycle and cell proliferation, from the reference peach genome^9^. Of these 231 genes, 192 were detected in the RNA-seq data and 44 were differentially expressed. We further selected 10 DEGs, including 8 DEGs that clustered together with expression levels higher in the round peach and 2 in the flat one at 0 DAFB (**Figure 7B**). In these 10 genes, five were associated with auxin, two with gibberellin, one with cell cycle, and two with cellulose. These 10 DEGs may act downstream of the flat shape gene to regulate the flat shape formation, because they are not located in the genome mapping site of flat shape gene.

After these two aspects analysis (**Figure 7**), we totally selected 33 DEGs that might participate in the process of flat shape formation, which were described in **Supplementary Table S4**.

### Validation of the Expression Patterns of Candidate Genes

**FIGURE 8:**
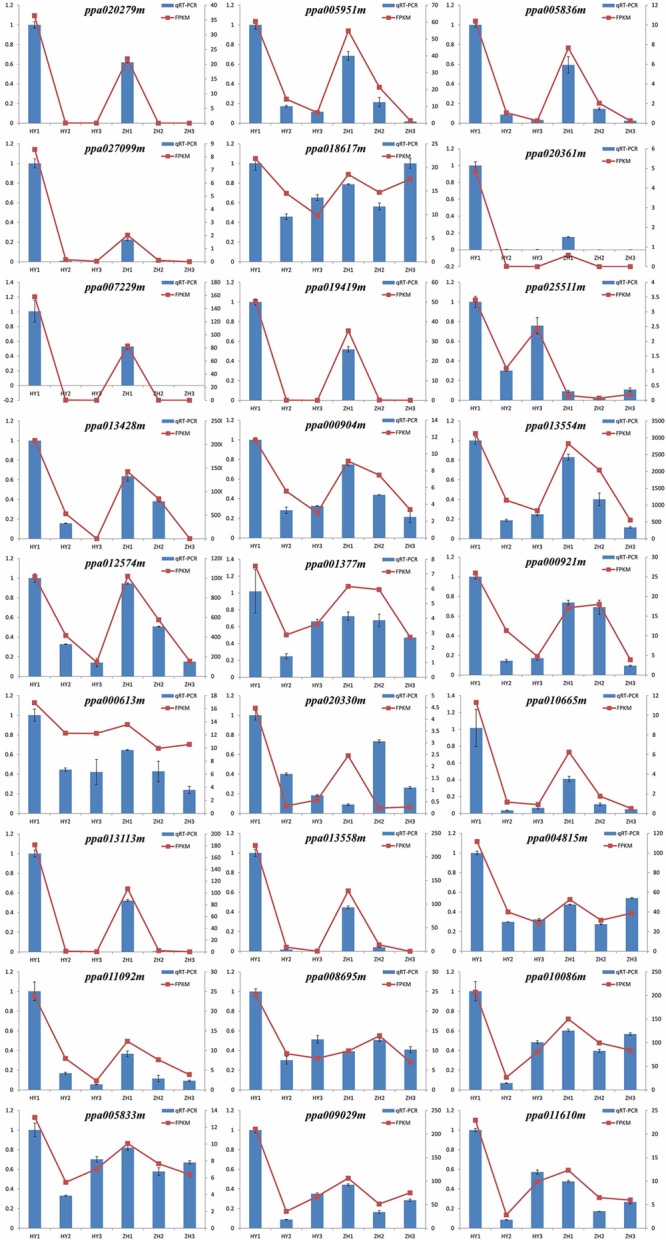
RT-PCR validation of expression levels of 28 DEGs identified by RNA-seq. The left vertical axis stands for the quantitative real-time polymerase chain reaction (qRT-PCR) and the right vertical axis stands for fragments per kilo-base per million mapped reads (FPKM).

To confirm the accuracy of the RNA-seq results and validate the expression patterns of candidate genes, qRT-PCR was performed with 27 selected genes which highly expressed in round peach at 0 DAFB. The relative expression levels of all the 27 genes were consistent with the results of RNA-seq analysis, which have confirmed the reliability of transcriptome data (**Figure 8**).

However, we selected these 33 DEGs merely through the heatmaps, but not the definite FPKM values. Therefore, there might be some genes that were unexpected in these 33 genes. We preferred to select genes which were differentially expressed at 0 DAFB and highly expressed at 0 DAFB than the other two stages. According to the results of qRT-PCR and also FPKM value, the number of candidate genes was further narrowed down from 33 to 28. In this section, 28 genes were finally regarded as key candidate genes named *ppa013047m, ppa004372m, ppa007229m, ppa019149m, ppa020361m, ppa020279m, ppa010655m, ppa009029m, ppa014358m, ppa003438m, ppa025511m, ppa010086m, ppa006443m, ppa013113m, ppa008642m, ppa004815m, ppa013558m, ppa013554m, ppa013428m, ppa000904m, ppa011610m, ppa011092m, ppa020330m, ppa000613m, ppa008695m, ppa005951m, ppa005836m*, and *ppa027099m* (**Supplementary Figure S4**). Nineteen were in the vicinity of major QTL of flat shape and nine were regarded as downstream genes, indicating that multiple genes may be involved in flat fruit formation in peach (**Table 2**). Two of the 19 genes were LRR-RLK (leucine-rich receptor like kinases) genes, including *ppa025511m* which was reported to be responsible for flat shape recently (López-Girona et al., 2017) and *ppa000904m*. Four of the 9 genes were associated with the auxin signal pathway (*ppa020279m, ppa005951m, ppa005836m, ppa027099m*).

**Table 2:** Annotations and physical locations of 28 candidate genes.

Gene name	Scaffold ID	Transcript length	Transcript start	Transcript stop	Annotation
ppa013047m	1	1238	31569356	31570593	YLS8; catalytic (related to cell cycle)
ppa004372m	1	3182	33752297	33755478	GA3; ent-kaurene oxidase (related to GA)
ppa007229m	2	1853	20097432	20099284	*O*-methyltransferase (related to cellulase)
ppa019149m	2	872	20126962	20127833	*O*-methyltransferase (related to cellulase)
ppa020361m	4	1314	7983415	7984728	Oxoglutarate/iron-dependent oxygenase (related to GA)
ppa020279m	4	1485	4267574	4269058	Tyrosine decarboxylase (related to auxin)
ppa010655m	6	2322	24144840	24147161	Zinc finger, RING-type
ppa009029m	6	984	24208617	24209600	Vitamin B6 biosynthesis protein
ppa014358m	6	1720	24324445	24326164	Unknown protein
ppa003438m	6	4027	24359484	24363510	Oxidoreductase
ppa025511m	6	2253	24405493	24407745	Leucine-rich repeat/transmembrane protein kinase
ppa010086m	6	2306	24519485	24521790	Ferritin/DPS protein domain
ppa006443m	6	2232	24610001	24612232	Phosphoglyceride transfer family protein
ppa013113m	6	429	24688963	24689391	Wound-induced protein
ppa008642m	6	1908	24875554	24877461	Peroxidase, putative
ppa004815m	6	2676	24936145	24938820	Protein kinase, catalytic domain
ppa013558m	6	738	25070010	25070747	Plant lipid transfer protein
ppa013554m	6	998	25072621	25073618	Plant lipid transfer protein
ppa013428m	6	800	25088833	25089632	Plant lipid transfer protein
ppa000904m	6	3459	25123698	25127156	Leucine-rich repeat/transmembrane protein kinase
ppa011610m	6	891	25375084	25375974	Late embryogenesis abundant protein, LEA-14
ppa011092m	6	1451	25406456	25407906	Initiation factor 2B-related
ppa020330m	6	267	25670294	25670560	Unknown protein
ppa000613m	6	3216	25782632	25785847	Peptidase M24
ppa008695m	6	1174	25908728	25909901	ABC transporter-like
ppa005951m	7	2382	13460554	13462935	Peptidase M20 (related to auxin)
ppa005836m	7	2213	13447174	13449386	Peptidase M20 (related to auxin)
ppa027099m	8	966	14000119	14001084	Hydrolase (related to auxin)

### IAA Concentration in Flat and Round Peach

**FIGURE 9:**
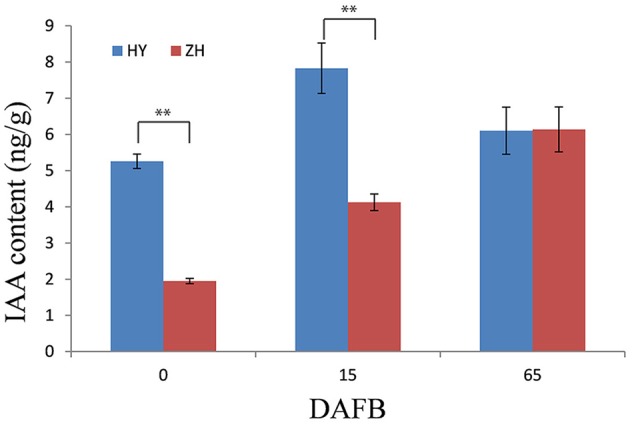
IAA content in flat and round peach. IAA content was measured in one round (HY) and one flat (ZH) peach cultivar using the same samples in RNA-seq analysis. ^∗∗^Represents the level of significance difference *P* < 0.01 in independent-samples *t*-test.

There were differences in IAA content between flat and round peach at early fruit development stages (0 and 15 DAFB) when the fruit shape began to form. The IAA content was much higher in round peach than flat one at the first two stages, while there was no difference at 65 DAFB (**Figure 9**).

## Discussion

### The Critical Cellular Factor That Determines Fruit Shape Variation

In this study, the cellular basis of flat and round peaches was investigated. Among the different size of fruits, cell number plays an important role in fruit size, such as in peach (Scorzal et al., 1991), blueberry (Johnson and Malladi, 2011), sweet cherry (Olmstead et al., 2007), apple (Denne, 1960), and tomato (Cheniclet et al., 2005). In peach, fruit growth shows a double-sigmoid pattern with cell production from full bloom to pit hardening and cell enlargement during the final period (Coome, 1976). Pits of peach cultivars examined in the present study began to harden at 35 DAFB.

In this study, we found that cell number in vertical direction determined peach flat and round fruit shape during early fruit development. This result supported the finding that peach flat shape was determined in the early stages of flower development (Dirlewanger et al., 1998). Cell number also determined fruit size in large and small fruits in peach (Scorzal et al., 1991). Based on these studies, we could find that cell number played a vital role in peach fruit development, including fruit shape and size. In peach, there are some other fruit shape types, such as oval shape. Whether these shape types are also determined by cell number remains unknown. And these results may provide some meaningful information to uncover other fruit shape types.

### Genetic Mechanisms That May Control Flat Shape Formation

Fruit shape formation is one of the most important processes of fruit development. For tomato, as the model fruit for fruit shape research, four fruit shape genes have been identified and named as *OVATE* (Liu et al., 2002), *SUN* (Xiao et al., 2008), *FASCIATED* (Cong et al., 2008), and *LOCULE NUMBER* (Munos et al., 2011). On the basis of their functional annotations and coding sequences, we did not find genes belong to these types in the 28 identified candidate genes, which indicated that there might have different mechanisms in the regulation of fruit shape formation between tomato and peach. However, their common characteristic is that cell number or size plays important roles in fruit development. In tomato, the gene *SUN* has functions in cell production, which results in a slender fruit phenotype (Wu et al., 2011) and microscopic analysis demonstrated that the higher pericarp thickness was not due to a larger number of cells, but to the increase in cell size (Su et al., 2014). Multiple quantitative trait loci for fruit size in tomato were associated with cell proliferation (Bertin et al., 2009). In apple, there were two genes associated with cell proliferation may result in vertical fruit development (Dash and Malladi, 2012). In crops, *WTG1* determines grain size and shape by influencing cell expansion (Huang et al., 2017). In cucumber, larger fruits have more cells and much larger cell area (Yang et al., 2013). No significant relationship was found between fruit diameter and cell area in Rabbiteye Blueberry genotypes (Johnson and Malladi, 2011). Similarly, increase in cell division has caused variation in fruit size in Japanese pear (Zhang et al., 2006). These studies have shown that genes regulating cell proliferation and expansion had a vital role in fruit shape and size, which support the finding of the present study. Data from this study indicate that mechanisms that regulate cell proliferation in the early period of fruit development determine the final fruit shape variation.

Recently, in our previous study, we discovered a candidate gene (*PpCAD1*) for flat shape in peach, which is annotated with CONSTITUTIVELY ACTIVATED CELL DEATH GENES 1 (Cao et al., 2016) and is expressed much higher in round peach than in flat one at fruit maturation stage. Although this allele can exactly classify flat and round peach, the nucleotide mutation in this gene is a SNP in the fifth intron of the gene and the main difference between flat and round peach is expression level at maturation stage; however, in the present study, we confirmed that the flat shape formation was mainly caused by the reduction of cell number in the vertical axis at early fruit development. In the present study, the samples were collected at fruit developmental stages. The qRT-PCR results of the present study showed that there were no differences in relative expression levels between flat and round peach at 0, 15, and 65 DAFB (**Supplementary Figure S5**), which indicated that *PpCAD1* might play important functions at maturation stage in regulating fruit development. The gene *PpCAD1* was identified through GWAS which was based on genotypes and phenotypes. However, in the present study, we focused on the gene expression level to identify genes related to fruit shape variation based on cellular proof. These two independent studies had different perspectives in identifying the flat shape gene. The high correlation between fruit shape and the SNP in *PpCAD1* made *PpCAD1* a more likely candidate, while, not like genes identified in the present study, the higher expression of *PpCAD1* in round peach at fruit maturation stage could not explain the fruit shape variation very well at early fruit development when the flat shape began to form. So, in our opinion, there might be no conflict between these two studies. The candidates of these two studies might have functions in the process of fruit shape formation together.

More recently, one gene named *ppa025511m* was identified to be co-segregated with flat shape in peach, because of a large deletion upstream of the gene and some nucleotide polymorphisms in the gene region (López-Girona et al., 2017). The primers for the flat shape marker provided by these authors could not distinguish the fruit types very well in our peach cultivars which were almost Chinese cultivars. As we known, peach originated in China and spread all over the world. Therefore, we believed that the diversities or amounts of the original flat peach cultivars that introduced into Europe may be too limited to produce a relative abundant genetic background, which may result in this unsatisfied result. Although we have found this gene in our transcriptome analysis, we believed that some other genes might be involved in flat shape formation. It has been reported that LRR-RLK genes have functions in plant organ regulation (De Smet et al., 2009; Mandel et al., 2014). There was a CLAVATA-WUSCHEL signaling pathway in the shoot meristem, with many LRR-RLK genes involved (Somssich et al., 2016). In our present study, we found two LRR-RLK genes (*ppa025511m* and *ppa000904m*) with an approximate physical distance 700 Kb. The *ppa025511m* itself may not be the flat shape gene, while these two genes may involve in flat shape formation together or there may be other genes involved in it. Therefore, we still could not discard any of them. The limitation of transgenesis in peach made it quite difficult to validate which gene is the real flat shape gene.

In our opinion, the flat shape gene resides in the genome region alongside these two previously reported candidate genes. *PpCAD1* may have function in fruit development at maturation stage, while *ppa025511m* at early stage. Therefore, in this study, 76 DEGs located within 1 Mb up- or down-stream of the main GWAS locus for fruit shape (Cao et al., 2016) were detected, including 19 expected in the relative expression patterns. Forty-four DEGs associated with plant hormones (Fosket and Torrey, 1969), cellulose (Cosgrove, 2005), cell cycle (Malladi and Johnson, 2011) and cell proliferation (Gillaspy et al., 1993; Harada et al., 2005) were also identified in the whole genome, including 7 expected (**Table 2**). In this study, we totally provided 28 candidate genes. Further studies should be carried out to identify the flat shape gene and verify the mechanisms among genes, cells and fruit development.

### Downstream Regulators Contribute to Fruit Development

In addition to the unconfirmed flat shape gene, some other downstream genes would participate in fruit shape formation. The gene (*ppa013047m*) annotated as yellow leaf senescence which has function in leaf senescence through cell cycle regulation (Yoshida et al., 2001) was highly expressed in flat peach at 0 DAFB, which might result in cell number reduction. Plant hormones have a major influence on plant growth. In a recent study, one WOX-like gene had functions in regulation of leaf width and stem thickness by enhanced cell proliferation in transgenic rice and *Brachypodium* and altered cytokinin homeostasis (Wang et al., 2017). In the present study, we did not find cytokinin-related genes, but four genes involved in auxin signal pathway (*ppa005951m, ppa005836m, ppa027099m, ppa018617m*). *ppa005951m and ppa005836m* were annotated as Peptidase M20 (Woodward and Bartel, 2005; Simunovic et al., 2011), *ppa027099m* and *ppa018617m* as hydrolase (Davies et al., 1999; Rampey et al., 2004). In peach, IAA has also been found to play a substantial role in fruit development, such as fruit ripening and softening (Tatsuki et al., 2013; Pan et al., 2015; Tadiello et al., 2016). In this study, the IAA concentration was higher in round peach than flat one at 0 and 15 DAFB when the fruit shape, which suggested that IAA might participate in the fruit shape formation in peach. Overall, the process of fruit shape formation is complicated, the results of the present study might aid other researchers to further illustrate the flat shape formation in peach.

## Accession Codes

Sequence data have been deposited in the NCBI Short Read Archive (SRA) under accession SRP116734. All other relevant data contained within the paper are available in Supplementary Files.

## Author Contributions

LW and KC designed the experiments. JG and YL analyzed the data. J-LY suggested the histological experiment. JG, YL, and QW prepared DNA and RNA samples. JG performed gene expression and paraffin section analyses. GZ, WF, XW, CC, LG, and TD prepared the materials. JG prepared the manuscript. J-LY and CD revised the manuscript.

## Conflict of Interest Statement

The authors declare that the research was conducted in the absence of any commercial or financial relationships that could be construed as a potential conflict of interest.

## Footnotes

1 http://imagej.nih.gov/ij/

2 https://www.rosaceae.org/node/355

3 https://www.rosaceae.org/

4 http://hannonlab.cshl.edu/fastx_toolkit/

5 http://www.ncbi.nlm.nih.gov/tools/primer-blast/

6 http://www.shimadzu.com.cn

7 http://www.appliedbiosystems.com.cn

8 https://www.rosaceae.org/node/355

9 http://pathways.rosaceae.org/pwy-search.shtml

## Supplementary Material

FIGURE S1 Family trees of the four examined cultivars.

FIGURE S2 Comparison of cell size and number between flat and round peach in cheek diameter.

FIGURE S3 Linear regression analysis of fruit cheek diameter, cell size, and cell number at fruit maturation stage.

FIGURE S4 The expression level heatmap of 28 candidate genes. Red color bands show high expression levels. Blue color bands indicate low expression levels.

FIGURE S5 The relative expression level of *ppa003772m*. Blue columns stand for relative expression levels of round peach. Red columns stand for relative expression levels of flat peach. The horizontal axis shows three different stages during fruit development (S1: 0 DAFB; S2: 15 DAFB; S3: 65 DAFB).

TABLE S1 Genotyping of 72 peach cultivars using the primers IndelS_R and FlatIn_F that are associated with *ppa025511m.*

TABLE S2 Primers used for qRT-PCR analyses.

TABLE S3 FPKM values, fold changes and annotations of 4165 DEGs.

TABLE S4 Description of 33 selected genes.
