# Supplementary figures and images for: Comparative Transcriptome and Microscopy Analyses Provide Insights into Flat Shape Formation in Peach (Prunus persica)
Source: Front Plant Sci. 2018 Jan 4;8:2215. doi: 10.3389/fpls.2017.02215 (PMC5758543; doi:10.3389/fpls.2017.02215)

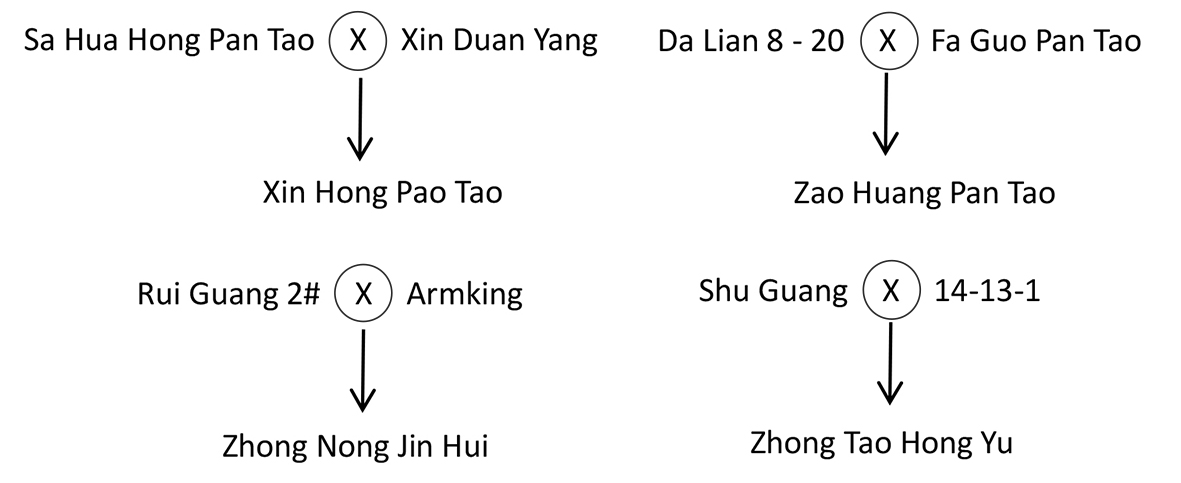

Supplement: FIGURE S1 — Family trees of the four examined cultivars. [file Image_1.JPEG]

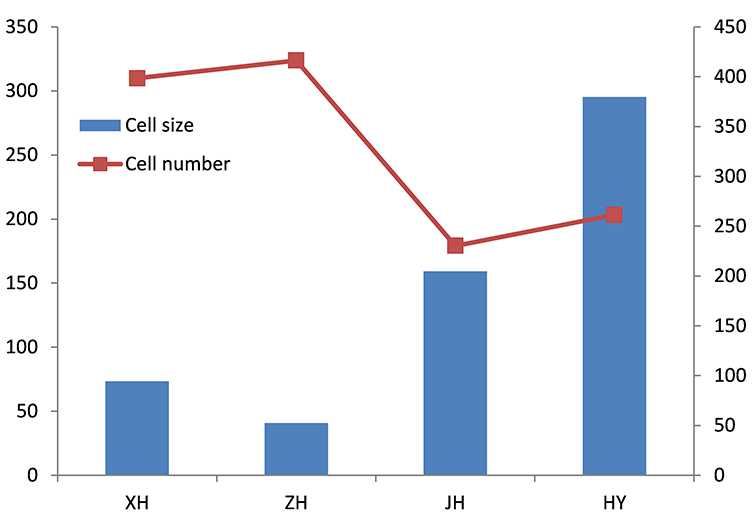

Supplement: FIGURE S2 — Comparison of cell size and number between flat and round peach in cheek diameter. [file Image_2.JPEG]

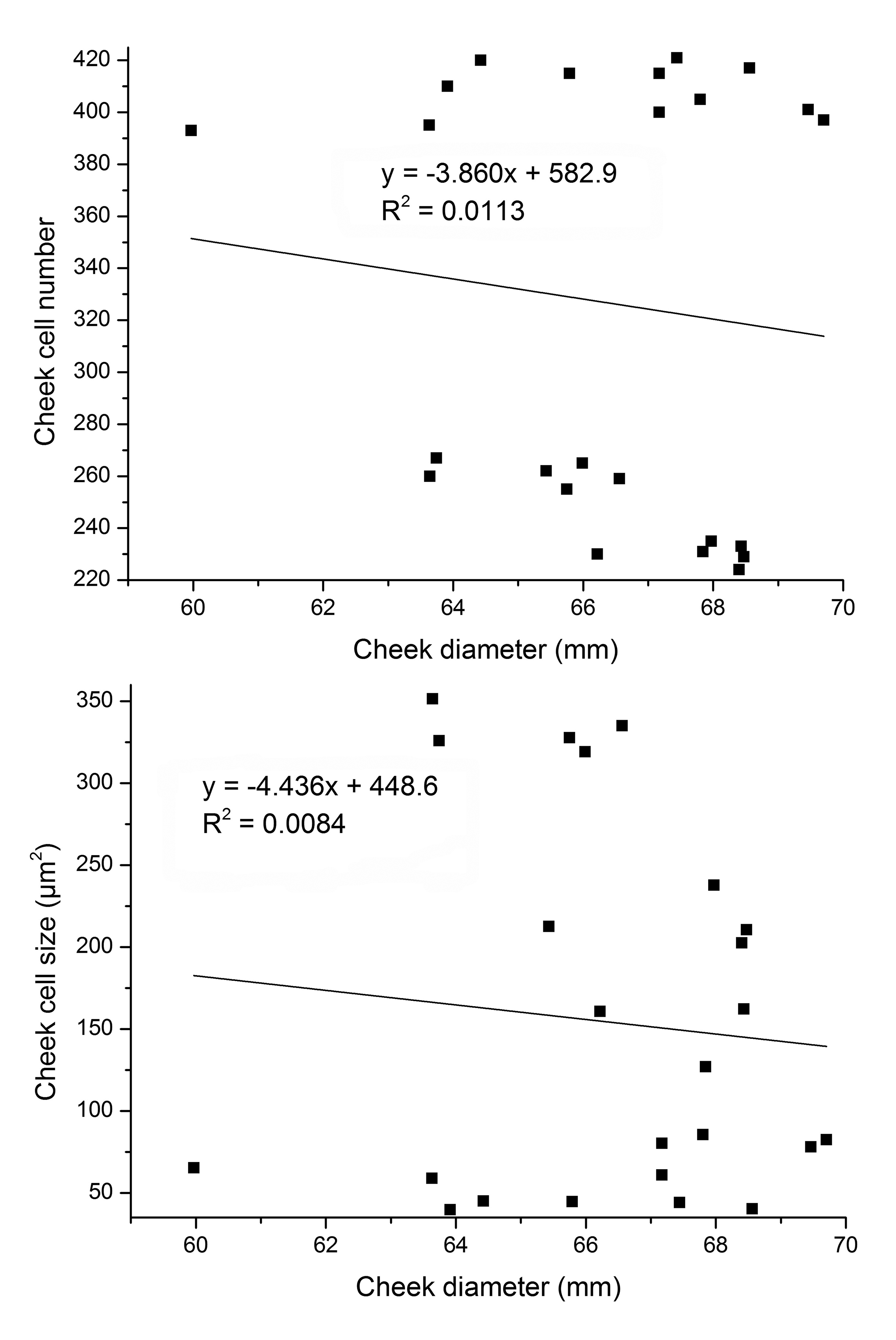

Supplement: FIGURE S3 — Linear regression analysis of fruit cheek diameter, cell size, and cell number at fruit maturation stage. [file Image_3.JPEG]

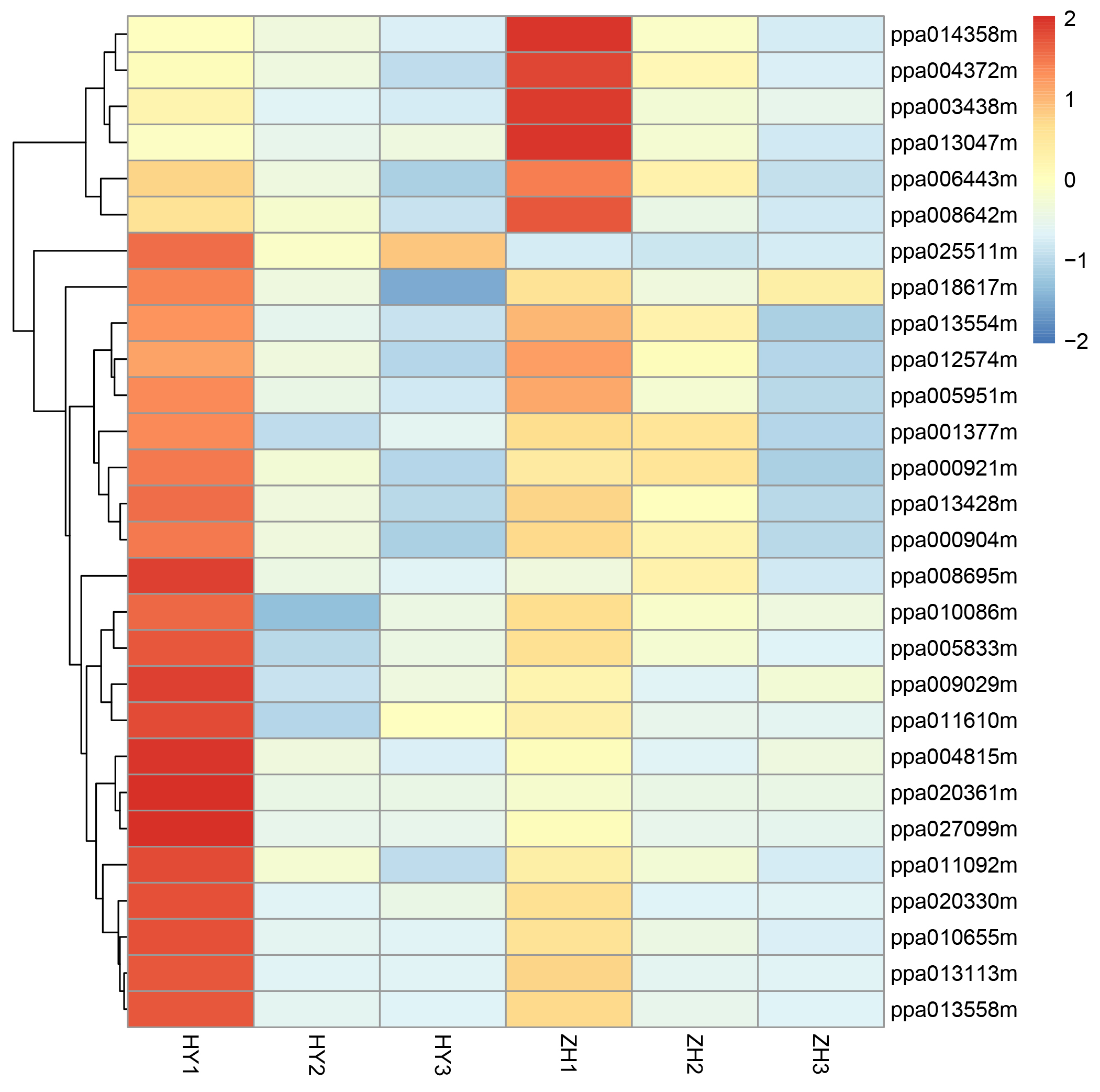

Supplement: FIGURE S4 — The expression level heatmap of 28 candidate genes. Red color bands show high expression levels. Blue color bands indicate low expression levels. [file Image_4.JPEG]

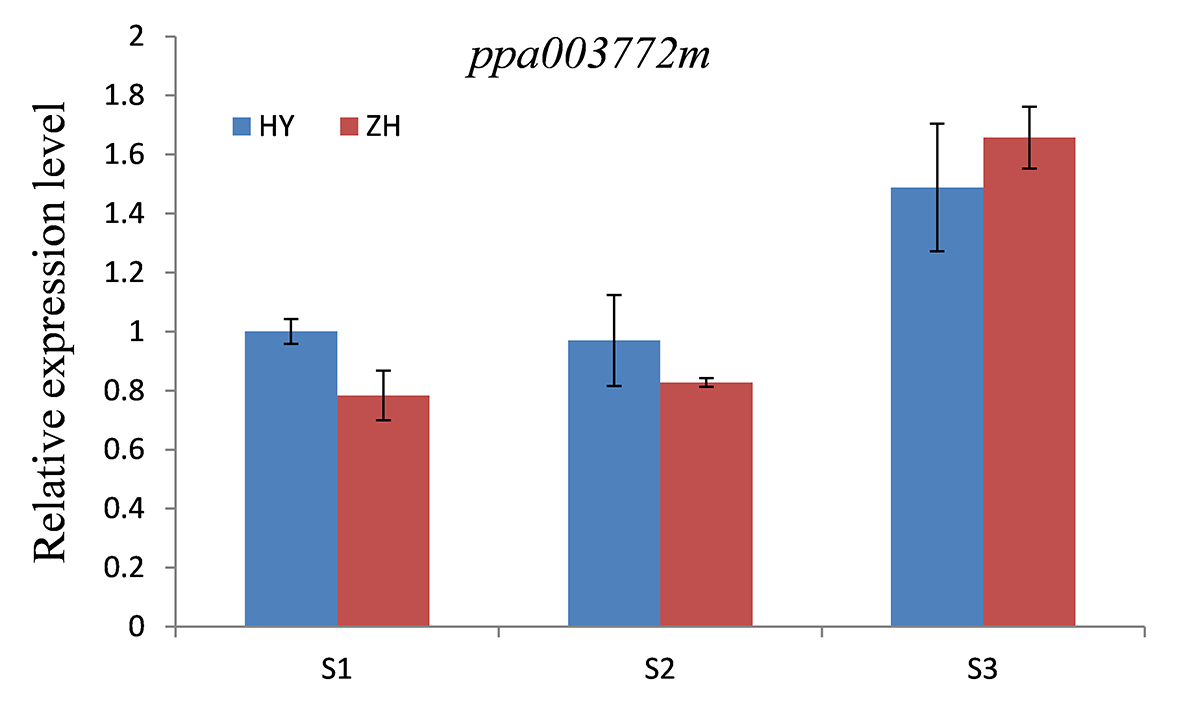

Supplement: FIGURE S5 — The relative expression level of ppa003772m. Blue columns stand for relative expression levels of round peach. Red columns stand for relative expression levels of flat peach. The horizontal axis shows three different stages during fruit development (S1: 0 DAFB; S2: 15 DAFB; S3: 65 DAFB). [file Image_5.PNG]
